# Insecticide resistance mechanisms with novel ‘kdr’ type gene mutations in the tropical bed bug *Cimex hemipterus*

**DOI:** 10.1186/s13071-019-3565-x

**Published:** 2019-06-21

**Authors:** Ranindra Punchihewa, W. A. Priyanka P. de Silva, Thilini C. Weeraratne, S. H. P. Parakrama Karunaratne

**Affiliations:** Department of Zoology, Faculty of Science, University of Peradeniya, Peradeniya, 20400 Sri Lanka

**Keywords:** *Cimex hemipterus*, Insecticide resistance, *kdr* type mutations, Resistance mechanisms, Tropical bed bug

## Abstract

**Background:**

The tropical bed bug, *Cimex hemipterus*, is a serious indoor public health pest in tropical regions causing intense physical discomfort and mental distress to humans. At present, the application of insecticides is the major control strategy. The present study was designed to evaluate the development of resistance and resistance mechanisms in *Cimex hemipterus* from Kandy district, Sri Lanka.

**Methods:**

The resistance status of the collected bed bugs was determined against the discriminative dosages of DDT, malathion, propoxur, deltamethrin and permethrin by conducting bioassays according to World Health Organization guidelines. Activities of insecticide metabolizing enzymes, i.e. esterases, glutathione S-transferases (GST) and monooxygenases, and the insensitivity of organophosphate/carbamate target site acetylcholinesterase (AChE), were evaluated by biochemical assays. Regions of the gene of the pyrethroid/DDT target site, the voltage-gated sodium channel regulatory protein (VGSC), were sequenced for possible *kdr* mutations.

**Results:**

Survival percentages of bed bug population were 71, 68 and 51% for DDT, malathion and propoxur respectively. KT_50_ and KT_90_ values, calculated using log-probit mortality curves for deltamethrin were 62.55 and 123.96 h, respectively. These values were much higher for permethrin where KT_50_ was 201.10 h and the KT_90_ was beyond the detectable range. Results were compared with previous values reported for the same population in 2002. Resistance to propoxur has increased significantly from 11 to 51% with about a 20-fold increase in the number of individuals with elevated esterase mechanism. No significant change has occurred in malathion and DDT resistance, in GST and monooxygenase activities, and in AChE sensitivity for the past 14 years. Six *kdr* associated mutations (Y/L995H, V1010L, I1011F, L1014F, V1016E, L1017F/S) and a non-*kdr* associated mutation (A1007S mutation) were found from the α-region of the *VGSC* gene. Out of the *kdr* type mutations, only L1014F has been reported previously form *C. hemipterus* while the others have been reported from other insects.

**Conclusions:**

The bed bug population has developed high resistance to propoxur with increased esterase activities. KT_50_ for deltamethrin and permethrin has increased 125- and 20-fold, respectively, over the period 2002 to 2016. To the authors’ knowledge, this is the first time that the possible involvement of a *kdr* type mutation in developing pyrethroid resistance in *C. hemipterus* has been shown in Sri Lanka.

## Background

Bed bugs have become a serious indoor public health pest around the world causing intense physical discomfort and mental distress to humans. In recent years, bed bugs have undergone a major resurgence in the number of infestations in the world [1]. Insecticide resistance is probably the key initiator of the bed bug resurgence, and resistant bed bugs have been disseminated worldwide through increased international travel. The tropical bed bug, *Cimex hemipterus*, is mainly confined to tropical regions and the common bed bug, *C. lectularius*, is found in temperate climates. However, both these species may appear beyond their normal ranges and inter-species mating may occur under both laboratory and field conditions, even occasionally producing a species hybrid [2, 3].

*Cimex* spp. are obligatory blood-feeders with the potential to vector pathogens. The most common clinical consequences arising from bed bug bites are direct cutaneous reactions [1]. Although their involvement in transmitting various disease agents such as bacteria (including *Rickettsia*), viruses, protozoans and nematodes were suspected in early literature, at present there is no evidence to suggest that bed bugs are involved in transmitting pathogens [4, 5]. Recent studies have reported that bed bugs are potential vectors of *Trypanosoma cruzi* [6, 7]. However, the physical stress, sleep deprivation and the mental health consequences arising from infestations have been reported to be serious [8]. Bed bug infestations have closed down hostels, hotels and even hospitals [1]. In the poultry industry, heavy infestations in poultry sheds have resulted in anemia and decreased egg production in chicken [9].

Non-chemical control mainly involves disposal of infested items, vacuuming and heat treatments [10, 11]. A variety of chemical formulations are available as powder dusts, fumigants and liquids against bed bugs. One major issue is the ineffectiveness of the insecticides labeled for bed bug control in the market and the main reason everywhere has been the failure of government insecticide registration authorities in evaluating the efficacy of new products based on insecticide resistance and resistance mechanisms of bed bug populations [12–14]. Overuse and misuse of insecticides for bed bug control has also led to increased selection pressure and thus to the rapid development of insecticide resistance [15]. Today, the vast majority of insecticides in use worldwide against bed bugs are pyrethroids. Resistance development against organochlorines, organophosphates (OPs), carbamates and pyrethroids has been documented for both species [1, 16, 17].

Although the mechanisms of resistance have been extensively studied in *C. lectularius*, only a few studies have been conducted with *C. hemipterus*. In insects, the increased metabolism of insecticides (metabolic resistance) through elevated enzyme activity and/or decreased target site sensitivity are the two major mechanisms of insecticide resistance. Metabolic resistance involves quantitative (through gene amplification, increased transcription and increased mRNA stability) and/or qualitative (through gene mutation) changes of insecticide metabolizing enzymes, i.e. esterases, glutathione S-transferases (GSTs) and monooxygenases [18, 19]. Point mutations in the voltage-gated sodium channel gene (*VGSC*) are responsible for the decrease sensitivity of the target sites for pyrethroids and DDT causing “knockdown resistance” (*kdr*). Two *kdr* type mutations have been previously reported from *C. lectularius* (V419L and L925I) and four have been reported from *C. hemipterus* (L899V, M918I, D953G and L1014F) [20]. Insecticide resistance and the underlying resistance mechanisms in the bed bug *C. hemipterus* collected from three districts of Sri Lanka were investigated in 2002 [21]. Here we report the development of increased resistance to propoxur with enhanced activity of esterases, and unusually high resistance to pyrethroids in one of these populations with six *kdr* type mutations, which have not (to the authors’ knowledge) been previously reported in *C. hemipterus*.

## Methods

### Bed bug collection

Bed bugs were collected from the previously described study sites [21] of Kandy district (7°15′N, 80°35′E; elevation 270–1060 m above sea level) in Sri Lanka during the period March to October of 2016. Samples were collected from 6 study sites and the maximum distance between these study sites was 2 km. Bed bugs were collected from crevices and cracks in the furniture and walls, and from bed mattresses using paintbrushes and forceps. Live bugs were brought to the laboratory and identified under a stereomicroscope. The identification of bed bugs was based on the key of Furman & Catts [22]. Blood-fed bed bugs were used for insecticide bioassays. Unfed bed bugs and the blood-fed bed bugs which were allowed to digest the blood meals, were snap frozen and stored at − 20 °C for biochemical and molecular assays.

### Chemicals and equipment

Chemicals were purchased from Sigma Chemicals UK (Haverhill, UK) unless otherwise stated. Permethrin (lot no. SZBD142XV), DDT (lot no. 360129B) and malathion (lot no. 352-138A) (97–99% pure) were a gift from the Liverpool School of Tropical Medicine, UK. Propoxur (lot no. SZBD302XV) was purchased from Bayer (Leverkusen, Germany). UVmaxELx 800^TM^ microtiter plate reader was from Molecular Devices, Bio-Tek, Winooski, VT, USA. The protein assay kit was from Bio-Rad (Dalkeith, UK).

### Preparation of insecticide papers

Insecticide impregnated papers were made according to the standard World Health Organization (WHO) insecticide impregnated paper method for the discriminating dosages specified by the WHO [23]. Only the discriminating dosage for DDT (2%) was available for *C. hemipterus* so those described for the common bed bug (*C. lectularius*) were used for malathion (5%), propoxur (0.8%), permethrin (0.25%) and deltamethrin (0.025%) [24]. Required concentrations were prepared by mixing the technical grade insecticides with a carrier; olive oil was used as the carrier for propoxur, malathion and DDT while Dow Corning 556 silicon fluid was used for permethrin and deltamethrin. Rectangles of Whatman No. 1 filter papers (12 × 15 cm) were impregnated with 0.7 ml of insecticide/carrier solution. An equal volume of acetone (0.7 ml) was used to ensure an even distribution of this solution on the paper. Papers were left at room temperature until the acetone had evaporated. They were then foil-wrapped and stored at − 20 °C.

### Bioassays

Adult bed bugs from the selected study sites were pooled together and subjected to insecticide bioassays *via* tarsal contact to insecticide impregnated papers using WHO mosquito bioassay kits [23]. Bed bugs were exposed to the insecticides as recommended by the WHO, i.e. 24 h for DDT and propoxur, 16 h for malathion, and continuous exposure for deltamethrin and permethrin. The insecticide chambers were fully covered using the insecticide impregnated papers and 10–15 bugs were exposed for one trial. After the fixed time periods, mortalities were recorded for DDT, malathion and propoxur while knock-down rates were recorded for deltamethrin and permethrin [24]. At least five replicates were carried out for each insecticide. Control insects were exposed to papers impregnated with carrier oil alone. Results were used only if the mortality/knockdown rate in the controls was < 20%. Control mortalities were adjusted for using Abbott’s formula [25].

### Biochemical assays

All the biochemical experiments were carried out according to the procedures outlined by the WHO [26] with slight modifications. Two hundred twenty-five randomly selected *C. hemipterus* adults were subjected to total protein, esterase, GST, monooxygenase and acetylcholinesterase assays in three replicates. Individual bed bugs were homogenized in 300 μl of ice-cold distilled water. An aliquot of 100 μl was taken for the acetylcholinesterase assay and the rest was centrifuged at 13,000× *rpm* for 2 min. The supernatant was used for esterase, GST, monooxygenase and protein assays [21].

### Detection of *kdr* mutations

DNA was extracted from 11 individuals of *C. hemipterus* using LIVAK extraction buffer [27]. Primers that had been designed to amplify the exon region between domain II S4 loop and domain II S5 of the gene code for VGSC regulatory protein to detect resistance-conferring mutations were used [28]. BBParaF1 (5′-AAC CTG GAT ATA CAT GCC TTC AAG G-3′) and BBParaR1 (5′-TGA TGG AGA TTT TGC CAC TGA TG-3′) were used to amplify fragment Ι and BBParaF3 (5′-CGA ATT GAA GCT GCC ATG AAG TTG-3′) and BBParaR3 (5′-TGC CTA TTC TGT CGA AAG CCT CAG-3′) were used to amplify fragment ΙΙΙ. The PCR reaction contained 2 μl of each primer (10 mmol), 14 μl of 2× GoTaq^®^ Green Master Mix, 5 μl of DNA template and 4 μl of Nuclease-Free Water (Promega, Madison, WI, USA). The mixture was amplified in a thermocycler for 30 cycles (95 °C for 1 min followed by 30 cycles of 95 °C for 30 s, 55 °C for 45 s and 72 °C for 60 s, and a final extension step at 72 °C for 7 min). An annealing temperature of 55 °C for 45 s was used to amplify fragment 1 and that of 58 °C for 45 s was used to amplify fragment 3. Products were viewed on an ethidium bromide stained 1% agarose gel. DNA sequencing was carried out using an automatic DNA sequencer (Series 3500, Applied Biosystems, Waltham, MA, USA) and the sequence was read for possible mutations. The sequences of gene fragments were aligned using ClustalW and compared using BioEdit software version 7.2. The GenBank database (NCBI) was used for blast searches with the *VGSC* gene of *Musca domestica* (accession number: AAB47604).

### Data analysis

SigmaPlot v.10 was used to obtain log-probit curves for insecticide concentrations against response knockdown rates (KT). KT_50_ and KT_90_ values for each bed bug population were estimated by regression analysis. Significant variations between elevated levels of enzyme activities of 2016 and 2002 bed bug populations and the percentage mortalities were performed using paired t-tests (Minitab v.15).

## Results

A total of 738 bed bugs were collected from the study sites and all were identified as *C. hemipterus*, from the ratio of length to width of the pronotum [22]. Percentage survivals resulting from DDT, malathion and propoxur bioassays are given in Table 1. No mortalities occurred in the control bioassay experiments. The bed bug population was resistant to all three insecticides tested. Comparatively, the highest level of resistance was shown to DDT while the lowest resistance was to propoxur. Resistance levels of the bed bug population, collected from the same area in 2002 to these three insecticides [21], are also present in Table 1 for comparison. Interestingly, resistance to malathion and DDT has not been changed for the last 14 years whereas propoxur resistance has increased from 11% to 51%. For permethrin, knock-down rates were much higher than that for the deltamethrin and a complete log-probit mortality curve could not be obtained as only about 50% of bed bugs were knocked down even after a one week of exposure to the discriminating dosage of permethrin. Pyrethroid bioassay results are presented in the Table 2 with the data of 2002 for comparison. No knock-downs were detected in the controls during the experimental period.

**Table 1:** Percentage survival of Kandy bed bugs after exposure to discriminating dosages of DDT, malathion and propoxur (*n *> 100 per insecticide). Exposure time for DDT and propoxur was 24 h whereas that for malathion was 16 h [24]. Data from the 2002 population [21] are also presented for comparison. Populations were statistically compared using two sample t-tests

Insecticide	2002		2016		Comparison	
	% survival	Status^a^	% survival	Status^a^	*t-*value^b^	*P*-value
DDT (2%)	71	Resistant	71	Resistant	0.132	1
Malathion (5%)	64	Resistant	68	Resistant	0.132	1
Propoxur (0.8%)	11	Possibly resistant	51	Resistant	4.532	0.0062*

^a^According to the classification of the WHO (2016): susceptible, > 98% mortality; possibly resistant, 80–98% mortality; resistant, < 80% mortality

^b^Degrees of freedom: 5 for all comparisons

*Significantly different

**Table 2:** Time (h) taken to knockdown 50% (KT_50_) and 90% (KT_90_) of the Kandy *Cimex hemipterus* population when exposed to discriminating dosages of deltamethrin and permethrin (*n *> 100 per insecticide) [24]. Data from the 2002 population [21] are also presented for comparison

Insecticide	2002		2016	
	KT_50_	KT_90_	KT_50_ (95% CI)	KT_90_ (95% CI)
Deltamethrin (0.025%)	0.5	1.0	62.55 (52.08–76.80)	123.96 (96.57–186.89)
Permethrin (0.25%)	10	24	201.10 (100.96–1653.00)	^a^

^a^Beyond the detectable level (see the text for details)

*Abbreviation*: CI, 95% confidence interval

**Fig. 1:**
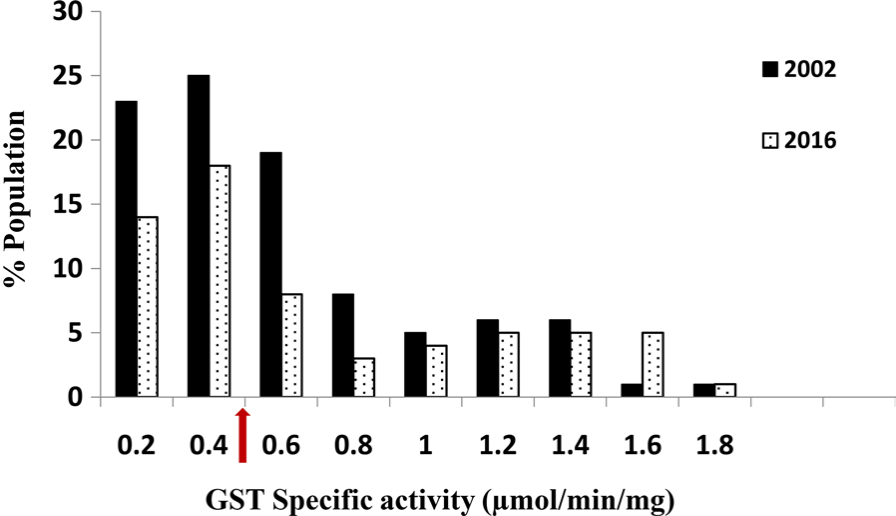
Activity profile of glutathione S-transferases (GST) of *Cimex hemipterus* population with chloro-dinitrobenzene as the substrate (*n* = 200). Data from the 2002 population [13] are also presented for comparison. The arrow on the X-axis represents the discriminating activity level (see the text for details)

**Fig. 2:**
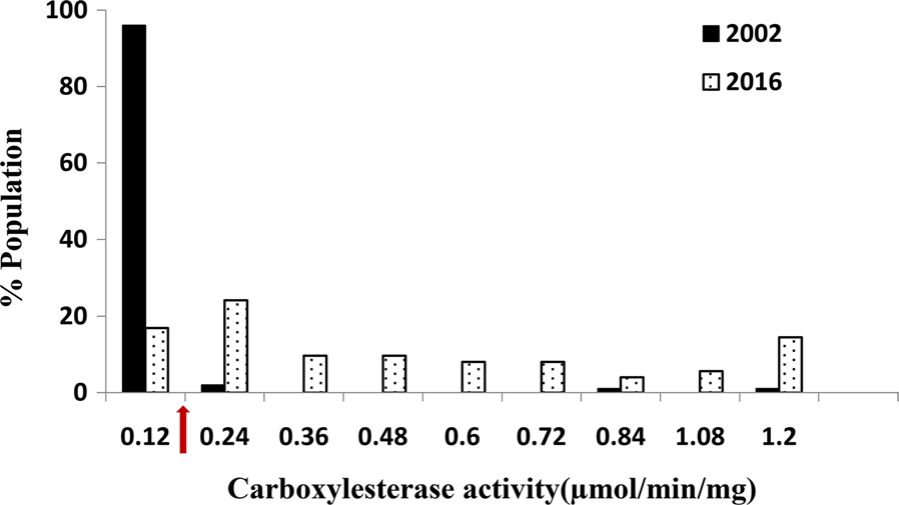
Activity profile of carboxylesterases of *Cimex hemipterus* population with *ρ-*nitrophenyl acetate as the substrate (*n* = 200). Data from the 2002 population [13] are also presented for comparison. The arrow on the X-axis represents the discriminating activity level (see the text for details)

**Fig. 3:**
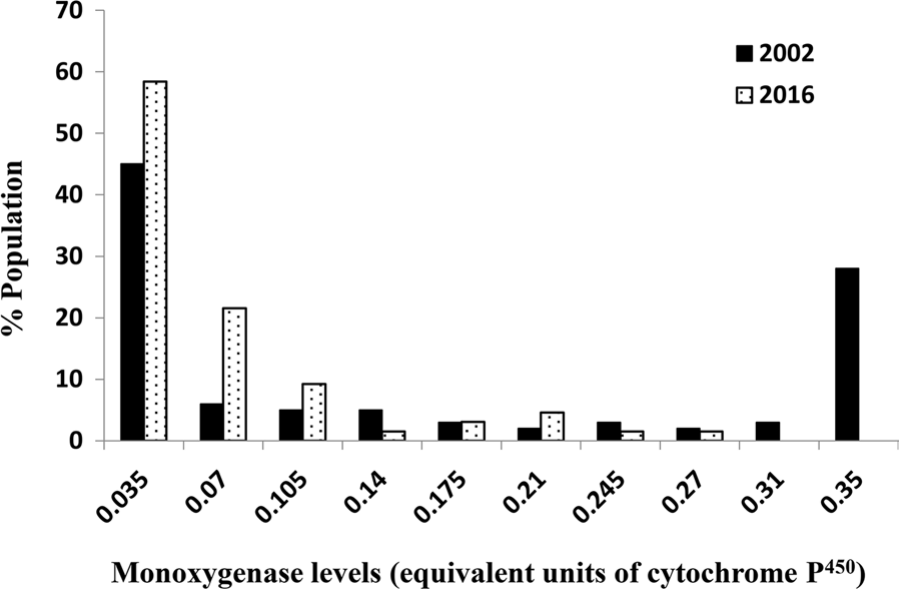
Estimates of monooxygenases (equivalent units of cytochrome P^450^) of *Cimex hemipterus* population (*n* = 100). Data from the 2002 population [13] are also presented for comparison. The arrow on the x-axis represents the discriminating activity level (see the text for details)

Results of the biochemical assays, conducted to assess the activity levels of insecticide detoxifying enzymes GSTs, carboxylesterases and monooxygenases in the bed bug *C. Hemipterus* population are shown in Figs. 1, 2, 3. Activity levels of these enzymes in the *C. hemipterus* population in 2002 are also presented to show the change over the years.

GST specific activity above 0.40 μmol min^−1^ mg^−1^ of protein was previously considered as a high activity which could contribute to metabolic resistance [21]. About 50% of the 2002 population and 32% of the 2016 population had relatively high GST activities, but the difference was not significant (*t*_(9)_ = 0.67, *P* = 0.80). The discriminating esterase activity was 0.12 μmol min^−1^ mg^−1^ of protein and only about 4% of the population had an esterase activity above this level for the 2002 population. In 2016, 83% of the population had an esterase activity more than the discriminating levels showing a significant increase in esterase activity (*t*_(8)_ = 1.88, *P* = 0.04). Monooxygenases in the bed bug populations were indirectly quantified by measuring bound haem [29]. Quantities below 0.35 equivalent units of cytochrome P^450^ have been reported from mosquitoes and ticks susceptible to this mechanism [30]. Monooxygenase activities of the 2016 population also fall within the susceptibility levels (Fig. 3) and the overall monooxygenase profiles between the two populations were not significantly different (*t*_(9)_ = 0.91, *P* = 0.19).

**Fig. 4:**
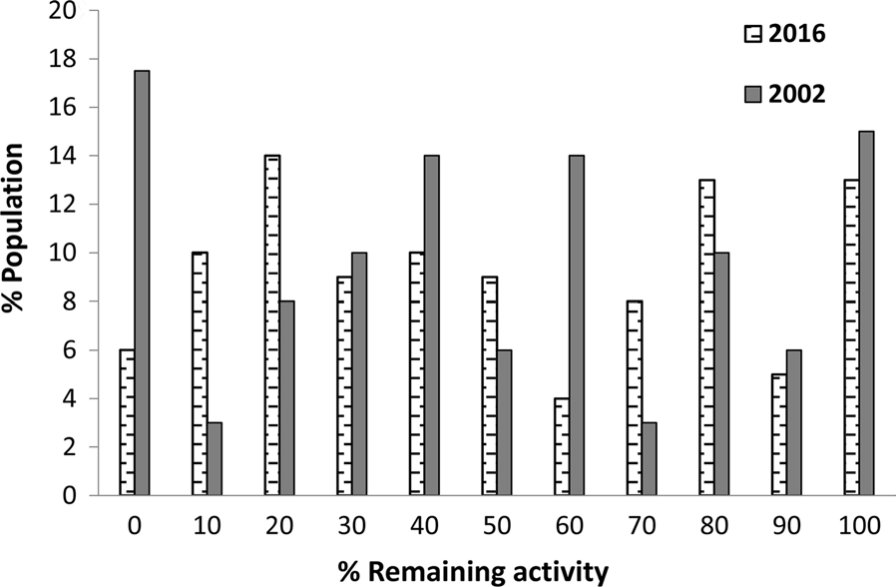
Percentage remaining activity of *Cimex hemipterus* acetylcholinesterases (AChEs) after inhibition with propoxur. Values were calculated as a fraction of the uninhibited activity of the same bed bug (*n* = 200). Data from the 2002 population [13] are also presented for comparison

The percentage remaining activities of OP and carbamate target site AChE of the *C. hemipterus* population is also presented with relevant data from the previous work (Fig. 4). According to WHO guidelines [26], results of this assay can be used to categorize the population into percentages of susceptible homozygous (SS, < 30% remaining activity), heterozygous (RS, 30–70% remaining activity) and resistant homozygous (RR, > 70% remaining activity) of altered or insensitive target site activity of AChE. Results showed that 30% of the bed bug population is susceptible homozygous, 32% of the population is heterozygous while 38% of the population is resistant heterozygous for the altered AChE mechanism in the present population of *C. hemipterus*. Respective values for the 2002 population were 25%, 45% and 30% (*t*_(10)_ = 0.17, *P* = 0.43).

**Fig. 5:**
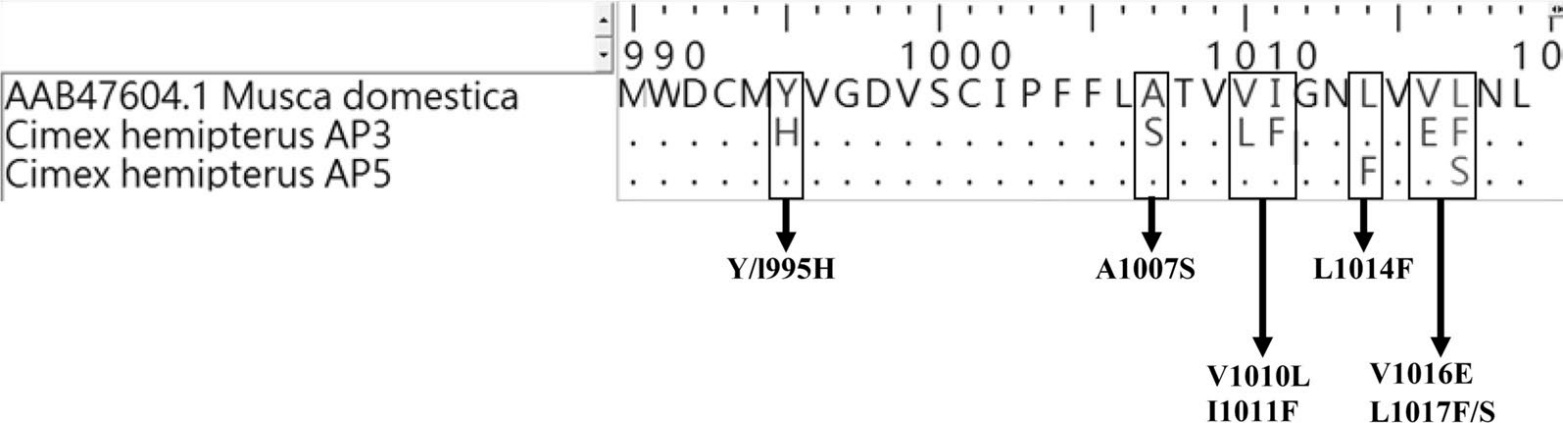
Gene sequences of the α-region of voltage-gated sodium channel of *Cimex hemipterus* aligned with that of *Musca domestica* (GenBank accession number: AAB47604) showing the mutations Y/L995H, A1007S, V1010L, I1011F, L1014F, V1016E, L1017F/S. Out of eleven, AP3 represents one individual sequence and AP5 represents ten individual sequences

DNA sequencing of fragment Ι and fragment ΙΙΙ of the α-region of the *VGSC* gene revealed the presence of mutations at Y/L995H, A1007S, V1010L, I1011F, L1014F, V1016E and L1017F/S in *C. hemipterus* (Fig. 5). *kdr* mutations L1014F and L1017S were found in 10 individuals (90.9%) and another six mutations were present in a single individual (9.1%). Out of these, A1007S mutation has been identified as having no association with the pyrethroid or DDT resistance [31]. All the other mutations have been identified as mutations of the *VGSC* gene facilitating *kdr* type insecticide resistance.

## Discussion

Early practices of bed bug control in Sri Lanka included applying boiling water and herbal extracts to small holes/crevices of the home furniture on which people sit or sleep. Insecticide treatment subsequently replaced these traditional measures. DDT was heavily used in insect control programmes until its cessation due to resistance development and environmental concerns in the mid-1970s in Sri Lanka. OPs and carbamates replaced DDT and pyrethroids were introduced in 1994. Pyrethroids became the major synthetic insecticide group used against the insect pests of both agricultural and health sectors, mainly due to their low mammalian toxicity and high efficacy. Bed bugs are also often controlled as a side-effect of mosquito control activities [32]. The development of DDT resistance in bed bugs as a response to anti-malaria indoor house-spraying campaigns [17, 32, 33] and pyrethroid resistance due to insecticide treated bed nets [34, 35] have been documented. DDT, Ops chlorpyrifos and dichlorvos, and the carbamate propoxur were the mainly used insecticides for bed bug control by householders in Sri Lanka until the introduction of pyrethroid formulations.

The present bioassay results show that the Kandy bed bug population has developed a very high level of pyrethroid resistance as a result of the increased use of pyrethroids. Resistance to pyrethroids has increased significantly over the period 2002 to 2016 and the time taken to kill 50% of the population has been increased 2.7-fold for deltamethrin and 15.1-fold for permethrin. *kdr* type mutations that could bring about pyrethroid resistance were not tested during the 2002 bed bug study. However, it is evident that several different mutations at the *VGSC* gene are responsible for the development of a high level of resistance to pyrethroids, mainly because the observed changes in metabolic enzyme activity levels are unlikely to provide such a resistance difference for pyrethroids although the increased esterase activity might be contributory to some extent. The contribution of other mechanisms, such as cuticular thickness, has been reported for pyrethroid resistance in *C. lectularius* [36].

Although DDT shares the same *VGSC* target site with pyrethroids, mutations occurring in the *VGSC* gene are unlikely to cause any DDT resistance, since the same magnitude of DDT resistance was shown in both 2002 and 2016 bed bug populations. Interestingly, the activity profiles of GSTs, which are mainly responsible for organochlorine metabolism, are similar in both populations. The persistence of a high level of DDT resistance in Sri Lankan insect populations long after its discontinuation has become a unique feature in almost all the insect populations in Sri Lanka [37–41]. This may be due to the fact that the mechanisms which were originally selected against DDT have later provided resistance to subsequently used insecticides or the mechanisms selected for the subsequently used insecticides provide cross-resistance to DDT.

Increased carbamate resistance can be correlated with increased esterase activities. However, malathion resistance, which had been developed through malathion carboxylesterase activity as a result of heavy malathion usage in anti-malarial activities in the past [21], remains unchanged. Insensitivity of the OP and carbamate target site AChE has not altered largely, and the monooxygenase levels have slightly decreased over the years.

The *kdr* type mutations of bed bug populations have been previously reported for *C. Lectularius* (V419L and L925I) and *C. hemipterus* (L899V, M918I, D953G and L1014F) [19, 20]. *Kdr* type mutations except for L1014F discovered in the *VGSC* gene during the present study are, to our knowledge, new records for the bed bug *C. hemipterus.* Thus, the six reported bed bug “*kdr*” mutations; Y/L995H, A1007S, V1010L, I1011F, V1016E and L1017F/S are new records for *C. hemipterus.*

From these six mutations, except A1007S mutations, all the other mutations have been previously reported as *kdr* type mutations causing pyrethroid and DDT resistance in *Anopheles* and *Aedes* mosquitoes. The Y/L995H mutation has been reported from Iranian *Anopheles culicifacies* [42, 43]. V1010L *kdr* mutation has been reported from 13 species of *Anopheles* mosquitoes from African, Asian and also from American continents [44, 45]. Even though the I1011V/M and V1016G are the commonly *kdr* type mutations [46, 47], Kandy bed bug population loci had slightly different amino acid substitutions as I1011F and V1016E. A1007S mutation has been previously reported from *An. funestus* from northern Cameroon [31], but with no association with *kdr*. The mutation I1017F has been reported from spiders, mites and ticks [48]. The present study reported the *kdr* mutation L1014F with a very high frequency. This mutation has been identified as one of the most common types of mutations present in many pyrethroid resistant insects, including *C. hemipterus* [20]. Hence, L1014F in Sri Lankan *C. hemipterus* also might contribute to pyrethroid resistance. However, further studies are needed to confirm and characterize the association between these mutations and the pyrethroid resistance of *C. hemipterus*.

The present study highlights that the bed bugs have developed a high level of resistance to all groups of synthetic insecticides. Resistant bed bugs are easily dispersed from place to place through infested cloths and other goods of humans. Efforts to control bed bugs using insecticides at the University of Peradeniya hostels, where about 5000 students reside, failed. It is important to look for alternative control methods for bed bugs. One such alternative is a properly managed heat treatment, which has been identified as a successful method of bed bug management [49–51]. Bed bugs die at temperatures ≥ 41 °C and the exposure time required shortens as the temperature increases [51, 52]. A study with dichlorvos in resin strips combined with heat and air treatments used in college dormitories has become successful in controlling abed bug population in the USA [53]. Future bed bug control programmes should be based on integrative approaches and future research should also be directed towards designing insecticides with novel target sites.

## Conclusions

The tropical bed bug *C. hemipterus* has developed a high level of pyrethroid and carbamate resistance through *kdr* type mutations and elevated esterases, respectively. The use of integrated approaches combining biological, physical and chemical methods should be emphasized in future bed bug control programmes.

## Data Availability

The datasets used and/or analyzed during the present study are available from the corresponding author upon reasonable request.

## Abbreviations

GST: glutathione S-transferase
AChE: acetylcholinesterase
VGSC: voltage-gated sodium channel
*kdr*: knockdown resistance
OPs: organophosphates
WHO: World Health Organization
KT_50_: time taken to knockdown 50% of the population
KT_90_: time taken to knockdown 90% of the population
